# Concomitant occurrence of primary gastric sarcomatoid carcinoma and giant gastrointestinal stromal tumor: a case report and literature review

**DOI:** 10.3389/fonc.2026.1757830

**Published:** 2026-02-27

**Authors:** Yujuan Dai, Jianhao Yang, Qiaozhen Li, LuSheng Wen, Xianying Chen, Yihui Wang, Lan Zhang, Dachao Chen

**Affiliations:** 1 Department of Oncology, The 909th Hospital, School of Medicine, Xiamen University, Zhangzhou, Fujian, China; 2 Department of Pathology, The 909th Hospital, School of Medicine, Xiamen University, Zhangzhou, Fujian, China; 3 Department of Obstetrics and Gynecology, The 909th Hospital, School of Medicine, Xiamen University, Zhangzhou, Fujian, China

**Keywords:** case report, diagnosis, gastric sarcomatoid carcinoma, gastrointestinal stromal tumor, pathology

## Abstract

**Background:**

Gastric sarcomatoid carcinoma (GSC) is a rare and aggressive malignancy, with only 16 cases reported in the English literature. Herein, we report an exceptionally rare case of synchronous primary GSC and gastrointestinal stromal tumor (GIST), a dual-tumor entity that has rarely been characterized in the existing literature.

**Case presentation:**

The patient was a 60-year-old man with GSC and GIST of the stomach who presented with dark stools and fatigue. Computed tomography (CT) imaging revealed gastric wall thickening and a large solid cystic mass adjacent to the gastric wall. Gastroscopic biopsy revealed a poorly differentiated carcinoma at the antrum-body junction. The patient underwent radical distal gastrectomy. Postoperative pathology indicated a sarcomatoid carcinoma (SC) of the greater curvature of the gastric body and an extraserous GIST. Postoperatively, adjuvant imatinib was administered. Two months later, multiple liver metastases were detected and confirmed by biopsy to be GSC metastases.

**Conclusions:**

This report describes a rare concomitant occurrence of GSC and GIST, with significant diagnostic challenges. In complex gastric tumors, vigilance for such a rare combination is essential, necessitating a thorough pathological evaluation for accurate diagnosis and individualized treatment. A misdiagnosis can lead to severe consequences.

## 1 Introduction

Gastric sarcomatoid carcinoma (GSC) is a rare epithelial-mesenchymal mixed malignant neoplasm, defined by the dual expression of epithelial (CK, EMA) and mesenchymal (vimentin) markers (1). Clinically, it displays highly aggressive behavior, with 60–80% of patients presenting with metastases at diagnosis and a median survival of just six months—markedly worse than conventional gastric cancer (2). With only 16 GSC cases reported in the English literature to date, we conducted a literature search of PubMed and Web of Science (WoS) using the terms *Gastric sarcomatoid carcinoma, Gastric carcinosarcoma, Gastric* sp*indle cell carcinoma, Sarcomatoid gastric carcinoma, and Gastric malignant neoplasm with sarcomatous differentiation* to synthesize existing clinical data. The search initially yielded 37 relevant articles, and 10 studies on GSC were selected following in-depth critical review, with their clinical characteristics summarized in **Table 1** (1, 3–11).

**Table 1:** Gastric sarcomatoid carcinoma cases in the current literature.

Author	Sex	Age (years)	Location	Size(cm)	Shape	Metastasis	Therapy	Prognosis
Robey-Cahherty SS (1)	M	78	Greater curvature	5	Polypoid	–	Surgery	45Mo.D
	F	57	Lesser curvature	5	Polypoid	–	Surgery	5Mo.D
	F	47	Gastroesophageal junction	5	Ulcerated	+	Surgery	8Mo.A
Nakayama N (3)	M	69	Remnant stomach	20	Polypoid	+	None	NA*
Sato A (4)	M	76	Remnant stomach	4	Polypoid	–	Surgery	7Mo.A
Chun-chao Zhu (5)	M	49	Distal stomach	14	Solid mass	–	Surgery	18Mo.D
Yi-yang Liu (6)	M	65	Lesser curvature	5	Ulcerated	+	Surgery	NA
	M	59	Remnant stomach	10.2	Ulcerated	+	Surgery	NA
	F	62	Cardia and Fundus	4.2	Mass	–	None	NA
	M	53	Fundus	1.4	Ulcerated	–	None	NA
	M	54	Cardia and Fundus	4	Ulcerated	–	Surgery	NA
You-peng Li (7)	F	54	Antrum	5	Ulcerated	–	Surgery	36Mo.A
Elghali MA (8)	M	80	Antrum	9.5	Thickening	–	Surgery	4Mo.A
Zhang Ao (9)	M	62	Fundus	10	Mass	–	Surgery	None
Yu-Jen hen (10)	M	70	Antrum and body	7.0	Ulcerated	+	Surgery	18Mo.D
Zhao R (11)	M	68	Cardia	10	Thickening	–	Surgery	25Mo.A
This case	M	60	Antrum and body	6	Ulcerated	–	Surgery	3Mo.D

*Patient died before surgical treatment. Mo, month; D, dead; A, alive.

Gastrointestinal stromal tumors (GIST) are the most common mesenchymal tumors of the gastrointestinal tract, accounting for 1% of all malignant gastrointestinal tumors (12). The stomach and small intestine are the most common primary sites of GISTs, while the colon, esophagus, and gastrointestinal tract are rare (13). The global average annual incidence of GISTs is 10–20 cases per million people with regional variations (14). While the coexistence of GISTs with epithelioid carcinoma is rare, their concomitant occurrence with GSC has been rarely documented in the literature. Herein, we describe a distinctive case double primary GSC and GIST complicated by liver metastasis that occurred 2 months after surgery. We also examine the clinicopathological challenges and treatment strategies.

## 2 Case description

**Figure 1:**
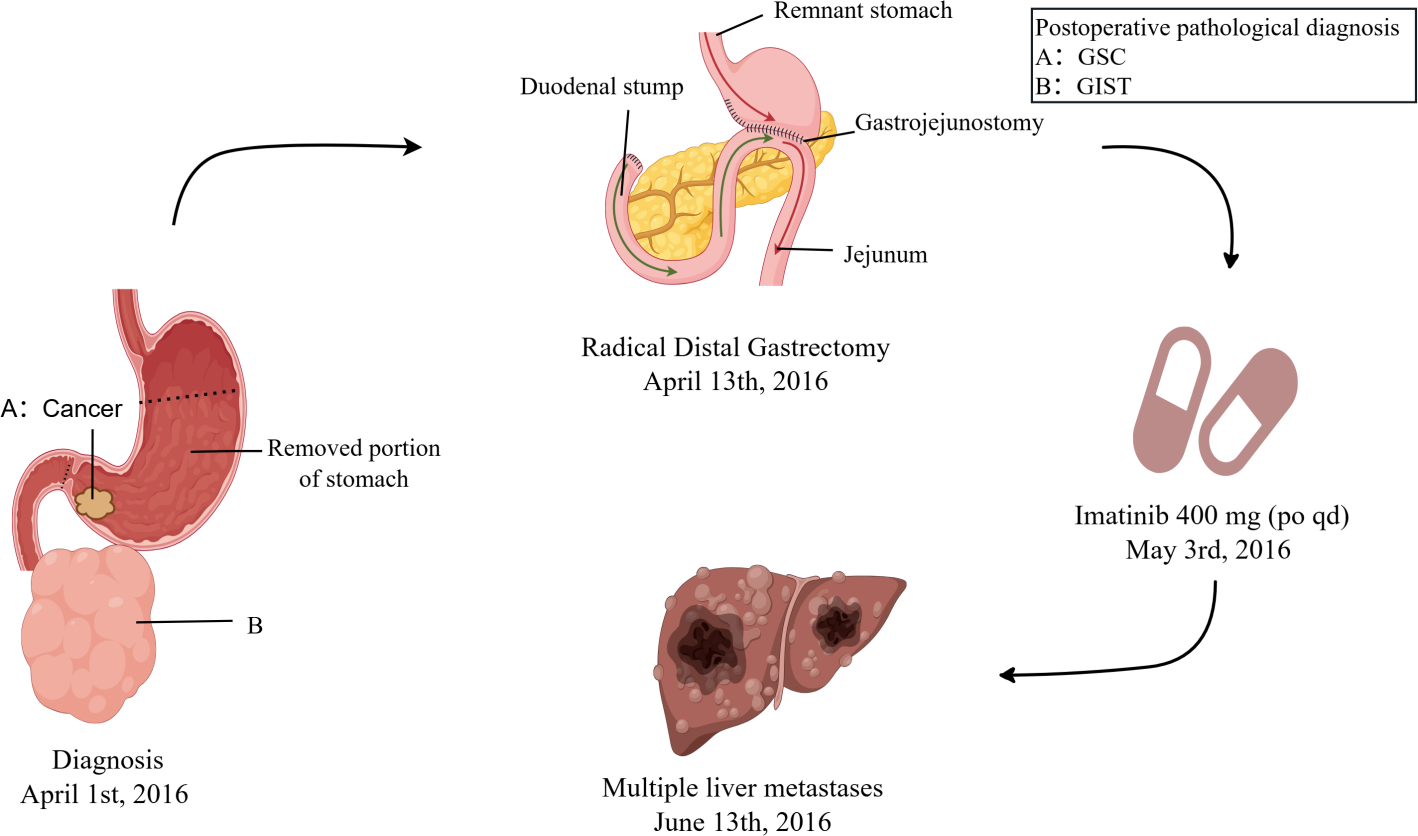
Timeline of clinical events for the patient with concurrent GSC and gastric GIST, including imatinib therapy and subsequent liver metastases.

**Figure 2:**
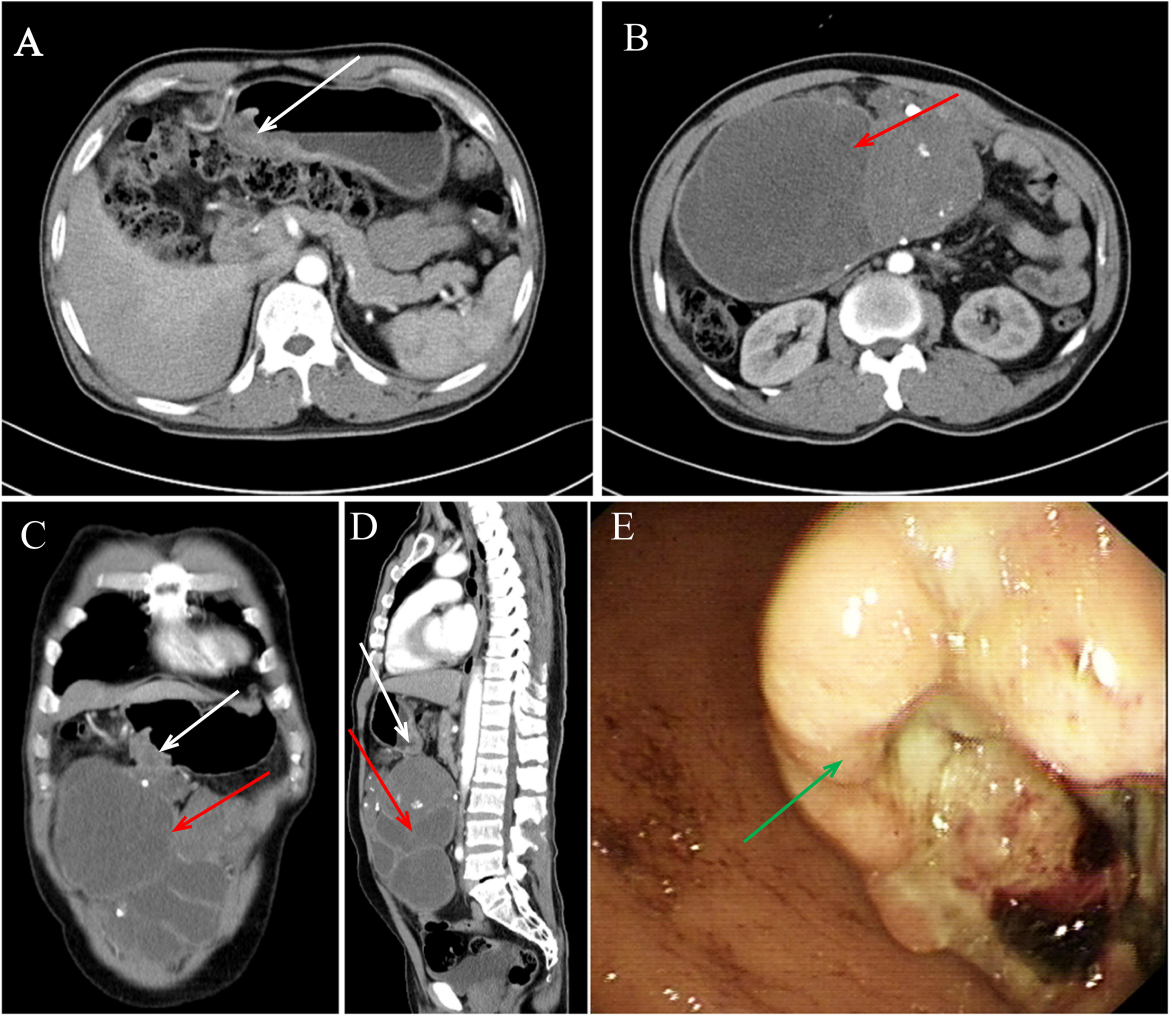
Imaging and gastroscopy findings. **(A–D)** Abdominal CT showed gastric wall thickening (white arrows) and a mixed solid-cystic mass (red arrows) along the greater curvature, with mild enhancement on contrast-enhanced imaging. **(E)** Gastroscopy confirmed a tumor at the gastric antrum-body junction (green arrow).

**Figure 3:**
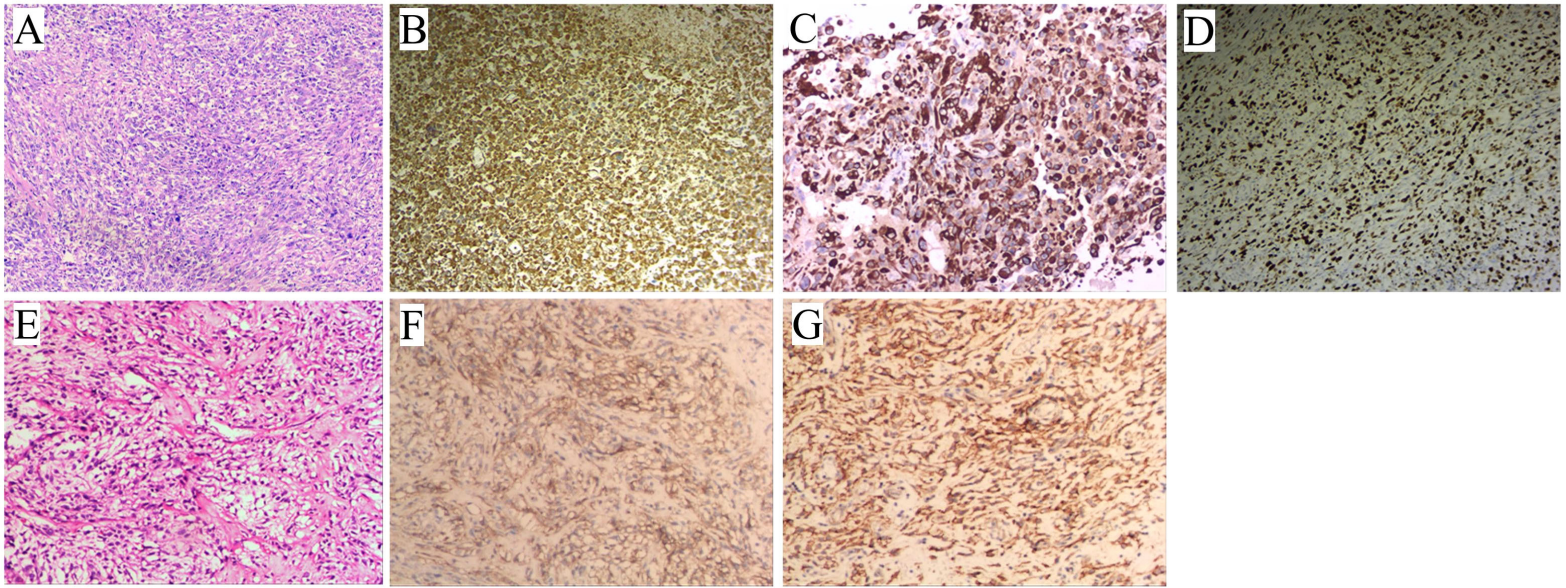
Histopathological and immunohistochemical features of GSC and GIST (×100). **(A)** GSC HE staining: Abundant pleomorphic spindle cells with disorganized arrangement and marked nuclear atypia. **(B)** GSC Vimentin IHC staining: Strong positive immunoreactivity, indicative of mesenchymal differentiation. **(C)** GSC Cytokeratin (CK) IHC staining: Strong positive expression, confirming epithelial lineage derivation. **(D)** GSC Ki-67 IHC staining: Diffuse positivity, indicating high tumor proliferation index. **(E)** GIST HE staining: Characteristic spindle cells arranged in fascicles, with eosinophilic cytoplasm and mild nuclear atypia. **(F)** GIST CD117 IHC staining: Diffuse strong positivity, a classic diagnostic marker of GIST. **(G)** GIST DOG1 IHC staining: Diffuse strong positivity.

**Figure 4:**
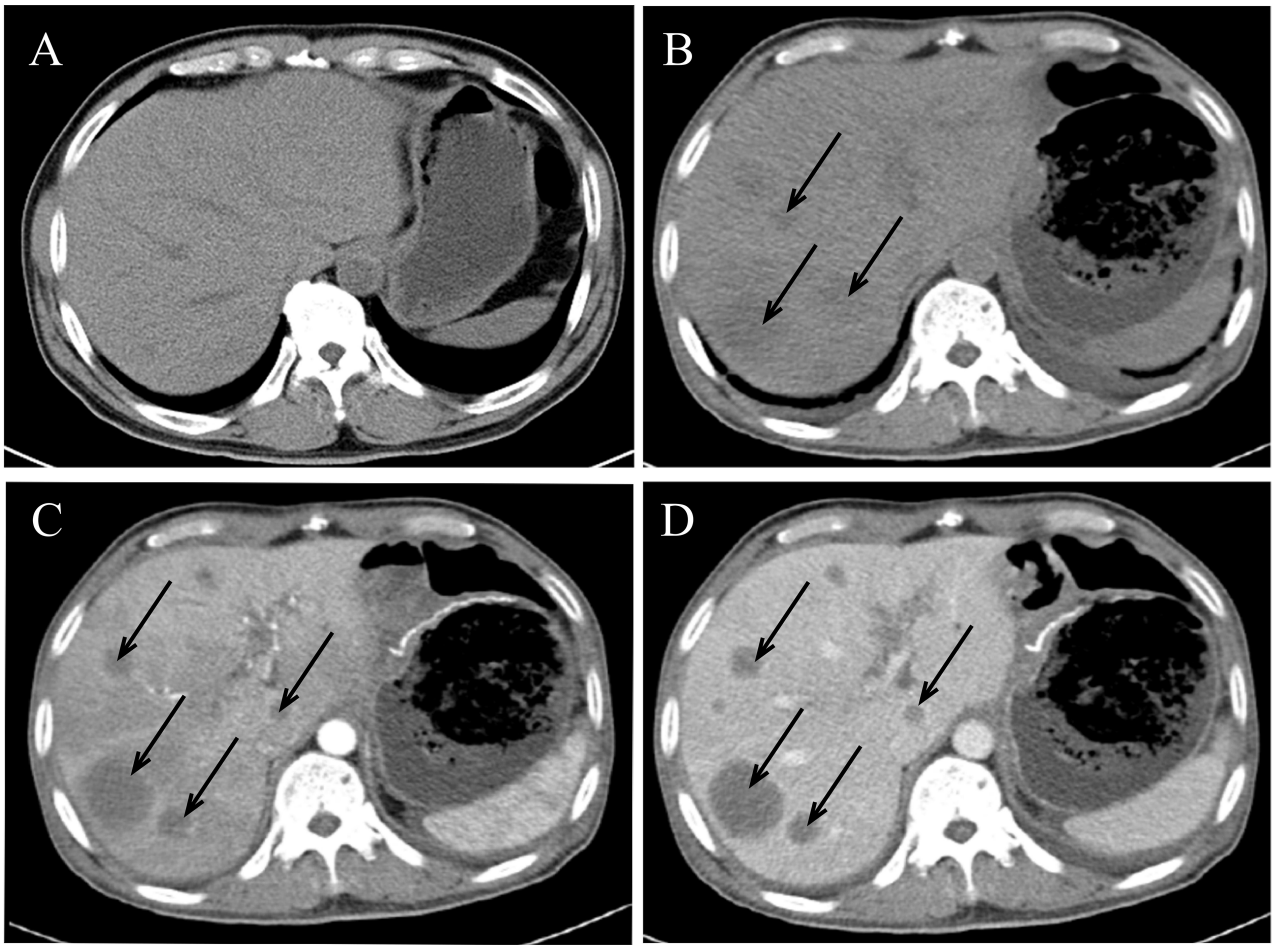
The pre- and post-operative CT findings. **(A)** The pre-operative CT scan shows no evidence of liver metastases. **(B–D)** The post-operative CT scans show the presence of multiple liver lesions consistent with metastatic disease (black arrows).

The patient’s detailed clinical diagnosis and treatment timeline (**Figure 1**) presents the key time nodes comprising preoperative evaluation, radical surgery, adjuvant targeted therapy, and liver metastasis identification. A 60-year-old male had no history of hypertension, diabetes mellitus, chronic hepatitis B, or family history of genetic disorders. He presented with melena, dizziness, and fatigue for over 10 days. Physical examination revealed abdominal distension and a palpable, large, well-defined, moderately firm mass in the middle and upper abdomen without tenderness or obvious pulsation. Contrast-enhanced abdominal computed tomography (CT) on admission revealed a large cystic and solid mass in the abdominal cavity, closely associated with the gastric antrum, which was considered to be either a teratoma or an ectopic thyroid with pathological changes (**Figures 2A–C**). Additionally, gastric antral wall thickening suggested a malignant tumor (**Figures 2A, C, D**). Gastroscopy findings indicated a mass at the gastric antrum-body junction, raising the suspicion of gastric cancer (**Figure 2E**). Pathological examination of endoscopic biopsy samples combined with immunohistochemistry (IHC) confirmed the diagnosis of poorly differentiated gastric carcinoma. The patient was fully aware of his clinical condition and the large abdominal mass. After being informed of the malignant nature of his gastric lesion, he expressed a strong desire for surgical intervention, aiming to alleviate melena, dizziness and fatigue and control disease progression. Having understood the potential risks of the proposed surgical and adjunctive treatment plans, he actively collaborated with the medical team to complete all preoperative assessments. A comprehensive preoperative evaluation revealed no obvious contraindications for surgery. Radical distal subtotal gastrectomy was performed under general anesthesia, alongside resection of the large abdominal tumor. Intraoperative examination demonstrated a 24 cm × 20 cm × 18 cm subserosal mass adjacent to the gastric greater curvature, exhibiting an intact capsule. Additionally, a firm mass sized 5 cm × 4 cm was palpated at the greater curvature of the gastric antrum. Tissue specimens resected intraoperatively were fixed in 10% neutral buffered formalin (NBF). Representative lesion tissues were dissected and placed into tissue processing cassettes, followed by a standard sequence of dehydration, clearing, and paraffin infiltration. The processed tissues were embedded using a paraffin embedding machine, and 3–5 μm-thick paraffin-embedded tissue sections were cut and mounted onto glass slides. After deparaffinization, the sections were subjected to either hematoxylin-eosin (HE) staining or IHC staining; all stained sections were finally coverslipped for microscopic examination (OLYMPUS BX43). HE staining of the adjacent area between GSC and GIST, clearly illustrating the histological boundary features of the two tumor components (**Supplementary Figure 1**). The HE staining morphological characteristics and IHC profiling of GSC (**Figures 3A–D**). The HE staining histological features and corresponding IHC phenotypes of the solid component of GIST (**Figures 3E–G**). The antibodies used and the results obtained are summarized in **Table 2**. Postoperative pathological findings are summarized as follows: (1), sarcomatoid carcinoma (SC) was located at the intraluminal aspect of the gastric greater curvature, infiltrating the entire gastric wall without breaching the adventitia, as confirmed by IHC; (2), GIST (extraserous mass) confirmed by IHC; (3), metastatic lesions were identified in the lesser curvature (2/31) and greater curvature (4/13) gastric lymph nodes, with no metastases in the pyloric lymph nodes (n=27). The GIST, located on the serosal surface and omentum of the gastric body greater curvature, presented as multiple nodules with a maximum diameter of 24 cm and ≤5 mitotic figures per 50 high-power fields. Tumor features (bleeding, necrosis, and cystic changes) suggested high malignant potential. The patient was prescribed adjuvant oral imatinib (400 mg/d) postoperatively. However, two months postoperatively, the patient developed abdominal distension, and subsequent CT revealed multiple liver metastases (**Figure 4**). Ultrasound-guided liver metastasis biopsy was performed, and pathological examination confirmed metastatic SC consistent with the primary tumor (**Supplementary Figure 2**).

**Table 2:** Primary antibodies, source, system and results in tumor cells.

Antibody (Abbreviation)	Clonality	Source	Result	
			Gastric mass	Subserosal Gastric mass
CK	Monoclonal	Maxin Biotech	+	–
Vim	Monoclonal	Maxin Biotech	+	+
CD34	Monoclonal	Maxin Biotech	+	–
CK19	Monoclonal	Maxin Biotech	+	–
CK56	Monoclonal	Maxin Biotech	–	–
LCA	Monoclonal	Maxin Biotech	–	–
CK8/18	Monoclonal	Maxin Biotech	–	–
TTF-1	Monoclonal	Maxin Biotech	–	–
S100	Monoclonal	Maxin Biotech	–	–
HMB45	Monoclonal	Maxin Biotech	–	–
CD117	Monoclonal	Maxin Biotech	–	+
SMA	Monoclonal	Maxin Biotech	–	+
Des	Monoclonal	Maxin Biotech	–	–
DOG1	Monoclonal	Maxin Biotech	–	+
CD56	Monoclonal	Maxin Biotech	–	–
Syn	Monoclonal	Maxin Biotech	–	–
Ki-67	Polyclonal	Maxin Biotech	80%	3%

Reagents: Ready-to-use formulations supplied by Maxin Biotech Co., Ltd. (Fuzhou, China), no dilution required prior to experimentation.

## 3 Discussion

GSC is an uncommon primary gastric tumor with unclear pathogenesis. Although GSCs are associated with *p53* gene mutations (15), the precise role of these mutations in GSC development remains uncertain. A key debate regarding the tissue origin of GSCs is whether they originate from common progenitor cells with multipotent differentiation or true epithelial-mesenchymal mixed lineage tumors (16). Accumulating mechanistic evidence supports the pivotal role of epithelial-mesenchymal transition (EMT) in driving stem cell formation and the pathogenesis of SC. Mani et al. demonstrated that activation of the EMT programs induces the generation of both normal and neoplastic epithelial stem cells (17). Lambert and Weinberg, in their comprehensive review, summarized that the majority of cancer stem cells (CSCs) in malignancies exhibit a quasi-mesenchymal phenotype with concomitant epithelial and mesenchymal traits. These cells acquire invasive capacity via partial EMT while retaining proliferative potential, acting as critical drivers of tumor recurrence and metastasis. Additionally, proinflammatory cytokines including TNF, IL-6 and IL-1β in the inflammatory microenvironment can directly activate the EMT program; in the tumor context, chronic inflammation can further modulate the EMT program to facilitate tumor metastasis (18). Notably, this inflammation-EMT regulatory axis affords a plausible mechanistic rationale for SC development in patients with a history of surgical trauma or chronic inflammation: surgical trauma typically elicits a localized, sustained inflammatory response, which in turn activates the EMT-associated signaling pathways. This persistent EMT activation may drive sarcomatoid differentiation of epithelial cells, ultimately culminating in the emergence of SC with aggressive biological behaviors. The link between inflammatory stimuli, EMT dysregulation, and sarcomatoid transformation thus lays a critical theoretical foundation for interpreting SC cases secondary to surgery or chronic inflammation (19).

The mechanism underlying the concomitant occurrence of GSC and GIST as dual primary neoplasms has not yet been fully clarified. Possible explanations include: (1), Common carcinogenic factors induce the malignant transformation of two distinct cell lineages (20); and (2), accidental biological events. Previous studies have shown that the incidence of secondary primary malignancies in patients with GIST ranges from 2.95% to 43% (21, 22), with gastric and colorectal adenocarcinomas being the most commonly reported associations (22, 23). However, to the best of our knowledge, this is a rare report of the concurrent occurrence of GIST and GSC.

The simultaneous occurrence of distinct primary tumors, particularly GSC and GIST, as in this case, presents a significant challenge for preoperative diagnosis. GISTs are typically located within the deeper muscularis propria or subserosa. In most reported cases of concurrent gastric adenocarcinoma and GIST, preoperative endoscopic biopsy often identifies only superficial epithelial carcinoma (such as adenocarcinoma), whereas deep-seated GISTs are difficult to sample (24). In this study, prior to surgery, gastroscopy revealed only an antral/somatic junction tumor. Biopsy indicated a poorly differentiated carcinoma, aligning with the epithelial components of GSC. However, the large GIST located outside the serosa went undetected, highlighting the concealed nature of deep GISTs. In concurrent cases, GISTs are typically small (<2 cm), with symptoms overshadowed by those of more prominent epithelial carcinomas (25).

GISTs typically manifest as well-delineated submucosal masses on imaging modalities, such as CT scans. Small GISTs tend to exhibit a homogeneous appearance with moderate contrast enhancement, whereas larger GISTs often display heterogeneous enhancement patterns attributable to necrosis, cystic degeneration, and hemorrhage, which may be accompanied by calcification and ulceration (26). CT findings in this case were initially misinterpreted as teratomas or ectopic thyroid lesions, and the characteristic radiographic features of GISTs were not accurately recognized, underscoring the complexity of diagnosing large and atypical GISTs using imaging modalities.

Surgical resection is the primary treatment for localized gastrointestinal stromal tumors (GISTs). Given the rarity of lymph node metastasis, extensive lymph node dissection is typically unnecessary (27). GIST prognosis is influenced by tumor location, size, mitotic count, and intraoperative rupture (28). Even with complete resection, approximately 40% of patients are at risk of metastatic recurrence, often in the liver and peritoneum (26). SC exhibits strong metastatic potential, and its prognosis is closely linked to the extent of the metastatic disease. Gastric SC generally shows poor response to conventional chemotherapy and radiotherapy, and there is no consensus on the optimal treatment approach. Patients with gastric SC typically have poor survival, with most experiencing relapse within one year (2). Studies on SC of unknown primary (SCUP) have shown significant chemotherapy resistance, with 77% (10 out of 13) of evaluable patients experiencing rapid disease progression and a median progression-free survival of only approximately two months when treated with first-line gemcitabine plus docetaxel (29). These findings strongly suggest that current chemotherapeutic regimens have limited efficacy against SC, underscoring the urgent need to explore new therapeutic strategies.

In cases of dual primary malignancies, prognosis is typically dominated by the more aggressive and progressive tumor (22, 25), as illustrated in the present case. Despite the large size of the GIST (24 cm), the patient developed liver metastases originating from the GSC, rather than the GIST, just two months after surgery, which is faster than the median time to metastasis for typical gastric cancer. GSC is generally an aggressive malignancy with rapid metastatic potential. In the present case, several factors may have contributed to the metastatic propensity of GSC. First, the tumor had invaded the full thickness of the gastric wall and was accompanied by pathologically confirmed regional lymph node metastasis. Second, Immunohistochemical (IHC) staining revealed overexpression of the Ki-67 proliferation index, which has been identified as an independent predictor of poor prognosis in cancer (30). Additionally, post-operative pathological examination revealed CD34 positivity, suggesting faster metastatic dissemination, potentially due to increased vascular invasion. Given the consensus that GSC responds poorly to adjuvant therapy, systemic adjuvant treatment was not administered in this case, which may have further facilitated rapid and widespread dissemination of the tumor.

This study offers a distinct and valuable contribution to the field of rare gastric malignancies by systematically documenting the clinicopathological, IHC, and follow-up characteristics of synchronous primary GSC and GIST—an exceedingly rare dual primary tumor entity with sparse documentation in the literature. First, it addresses the paucity of evidence regarding the coexistence of these two histogenetically divergent neoplasms (epithelial-derived GSC and mesenchymal-derived GIST), elucidating their adjacent anatomical relationship, distinct expression patterns of core biomarkers (e.g., GSC demonstrates positivity for CK/Vim with a high Ki-67 proliferation index of 80%; GIST is positive for CD117/DOG1), and the aggressive metastatic propensity of GSC. This furnishes critical diagnostic insights for distinguishing dual primary tumors from metastatic lesions or single malignant tumors with heterogeneous components. Second, the study underscores the clinical dilemma of missed diagnosis of deep-seated GISTs during preoperative assessment, epitomized by the 24cm extraserous GIST not identified on gastroscopy, thereby underscoring the necessity of utilizing multiple diagnostic approaches and intraoperative pathological assessment. Recognizing the inherent limitations of a single-case report, future research could amass cases via a multicenter registry, examine prognostic determinants of such dual primary tumors, and unravel the molecular drivers governing their synchronous development using next-generation sequencing (NGS) technology.

## 4 Conclusions

GSC is an exceedingly rare tumor, and its concurrence with GIST as double primary lesions is extremely rarely reported in the literature. This case highlights the significant diagnostic and treatment challenges associated with complexity. The rapid progression and poor prognosis are primarily due to the highly invasive nature of sarcomatoid carcinoma, which has substantial metastatic potential. Overexpression of Ki-67 and increased vascular invasion (CD34+) are likely underlying factors of its rapid metastasis.

## Data Availability

The original contributions presented in the study are included in the article/**Supplementary Material**. Further inquiries can be directed to the corresponding authors.

## Supplementary material

Supplementary Figure 1 Pathological exam. **(A)** Adjacent lesions of gastric stromal tumor (GIST) and gastric sarcomatoid carcinoma (GSC) (hematoxylin-eosin staining, ×40). **(B)** Gastric stromal tumor (GIST) component,fascicular spindle cells with eosinophilic cytoplasm and bland nuclei (hematoxylin-eosin staining, ×200). **(C)** Gastric sarcomatoid carcinoma (GSC) component, pleomorphic spindle cells, disorganized arrangement, marked nuclear atypia (hematoxylin-eosin staining, ×200).

Supplementary Figure 2 Liver puncture pathology. Tumor cells are distributed in sheets with obvious atypia.
