# Supplementary figures and images for: Concomitant occurrence of primary gastric sarcomatoid carcinoma and giant gastrointestinal stromal tumor: a case report and literature review
Source: Front Oncol. 2026 Feb 27;16:1757830. doi: 10.3389/fonc.2026.1757830 (PMC12982032; doi:10.3389/fonc.2026.1757830)

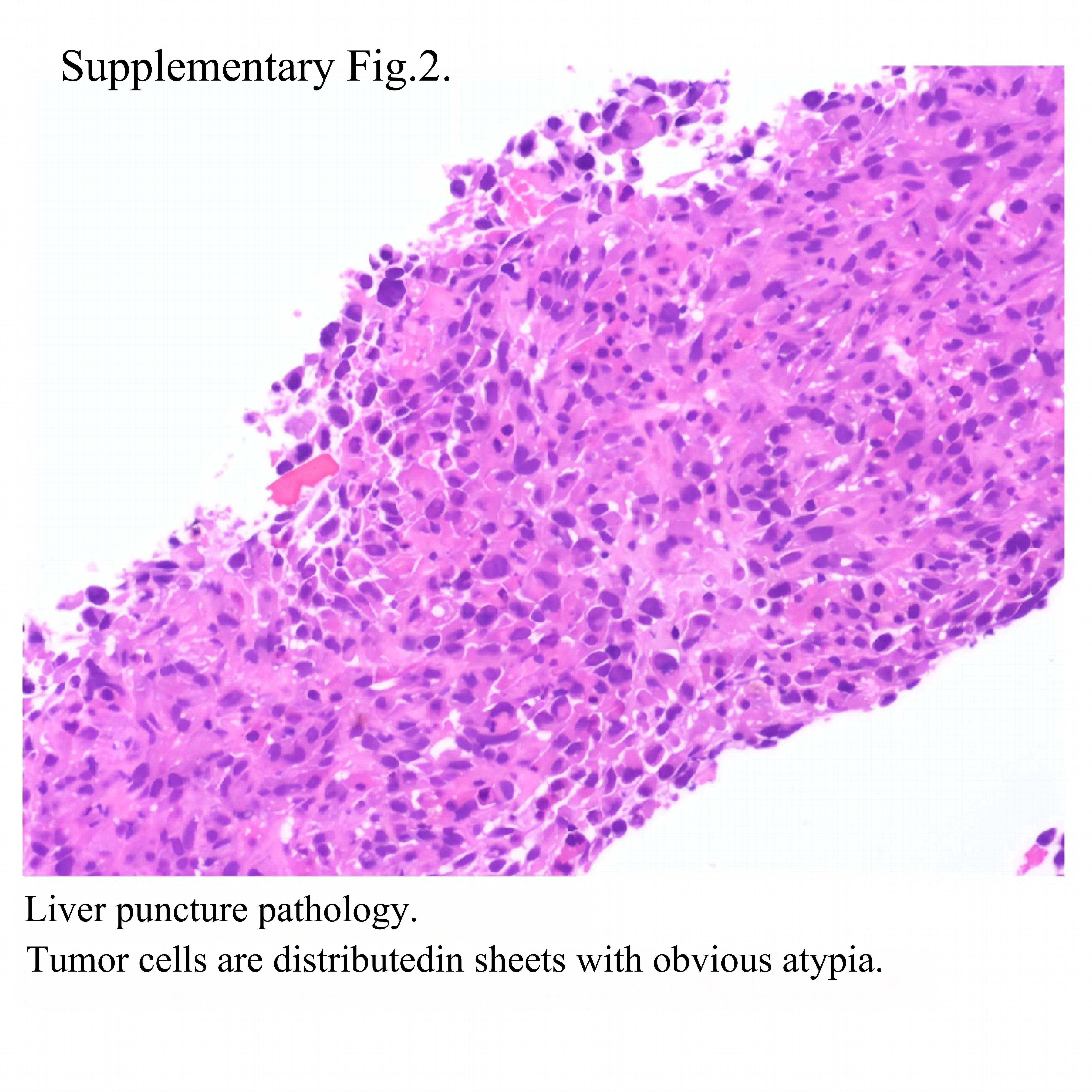

Supplement: Supplementary Figure 2 — Liver puncture pathology. Tumor cells are distributed in sheets with obvious atypia. [file Image2.jpeg]
